# DSPicable V: TuMV exploits a dual-specificity phosphatase to suppress MAPK signaling and host resistance

**DOI:** 10.1093/plcell/koaf238

**Published:** 2025-10-03

**Authors:** Rory Osborne

**Affiliations:** Assistant Features Editor, The Plant Cell, American Society of Plant Biologists; School of Biosciences, University of Birmingham, Birmingham B15 2TT, UK

Posttranslational modifications modulate protein function in all living organisms. The addition or removal of chemical moieties at specific amino acid residues, such as phosphorylation, acetylation, and ubiquitylation, dynamically regulates protein activity and turnover. Phosphorylation is an essential component of cell signaling cascades via MITOGEN-ACTIVATED PROTEIN KINASEs (MPKs). In the context of plant immunity, MPKs directly phosphorylate transcription factors that activate defense responses after microbial perception at the cell surface (Sun and Zhang 2022). To avoid autoimmunity and associated stress, however, it is critical that these signals be appropriately downtuned when they are no longer required. While activation of MPK cascades is well characterized, mechanisms describing their attenuation are less well understood.

Previous studies have suggested that plant and fungal atypical dual-specificity phosphatases (DSPs) deactivate MAPK signaling in rice and Arabidopsis in response to diverse phytopathogens (He et al. 2012). Seeking to understand how the MPK cascade is dampened after viral infection, Yameng Luan, Xue Jiang, and colleagues (Luan et al. 2025) investigated the role of Arabidopsis DSPs in response to turnip mosaic virus (TuMV). The authors first characterized the physiologic resistance of 5 *DSP1-5 Arabidopsis thaliana* mutants to TuMV, revealing that the *dsp4-2* mutation attenuated infection. Given that *DSP4* was upregulated in this line, the authors hypothesized that DSP4 negatively regulates viral immunity.

Taking a combined approach of confocal microscopy and quantitative ultracentrifugation, the authors next showed that YFP-DSP4 predominantly localizes to the endomembrane and that this localization likely depends on the prenylation of a cysteine residue near its C-terminus. Furthermore, activation of pattern-triggered immunity (PTI) induced the dissociation of DSP4 from the endomembrane system, suggesting that it might have a role in downstream immunity signaling. Indeed, corroborating previous evidence that DSPs interact with MPKs (Xin et al. 2021), the authors used BiFC and CoIP to show that DSP4 physically interacted with multiple MPKs. Focusing on the DSP4–MPK6 interaction revealed that membrane-bound DSP4 could dephosphorylate MPK6 in vitro. This activity was lost when mutations to the DSP4 prenylation site were introduced, leading the authors to hypothesize that membrane association is required for effective MPK6 interaction/dephosphorylation.

MPK signaling was then shown to be an essential component of viral immunity. TuMV infection induced the expression of 16 *MPK* genes and promoted the phosphorylation of MPK3 and MPK6. Viral challenge of *mpk6* knockouts revealed a lower rate of viral gene expression and infected leaf area as compared with wild type, which was further enhanced when these plants were complemented with *pMPK6::FLAG-4×Myc-MPK6*. However, concomitant repression of *MPK3* via viral-induced gene silencing in a *mpk6* knockout vastly decreased host resistance. In summary, the authors concluded that phosphorylated MPK3/6 redundantly suppressed TuMV infection.

Of course, no defense is foolproof, and pathogens regularly co-opt host machinery to facilitate their infection. Encoded in the TuMV genome is protein 3 (P3), a known membrane virulence factor that disrupts Rubisco activity (Lin et al. 2011). P3 was shown to interact with DSP4, which altered DSP4 localization and induced the formation of membrane condensates, suggesting that P3 might reduce the dephosphorylation activity of DSP4. However, an in vitro phosphorylation assay revealed that the opposite was true and that P3 enhanced DSP4 dephosphorylation of MPK6.

This conflicting result suggests either that the relocalization of DSP4 by P3 is an artifact induced by agroinfiltration (which itself induces PTI) or that these membrane condensates remain part of the endomembrane system and concentrate functional DSP4. Some evidence to the latter was demonstrated, as coexpression of both proteins increased the amount of the DSP4 associated with the endomembrane. While clearly showing that DSP4 attenuates an overactive immune system by dampening MPK6 activity, the mechanism of P3-mediated disruption to this process remains unclear. The authors proposed model (Fig.) suggests that P3 reduces the dissociation of DSP4 from the endomembrane system, increasing the dephosphorylation of MPK6 to impair host resistance. Further characterization of the MPK6–DSP4–P3 relationship, especially in vivo, will be required to decode this mechanism.

**Figure.**
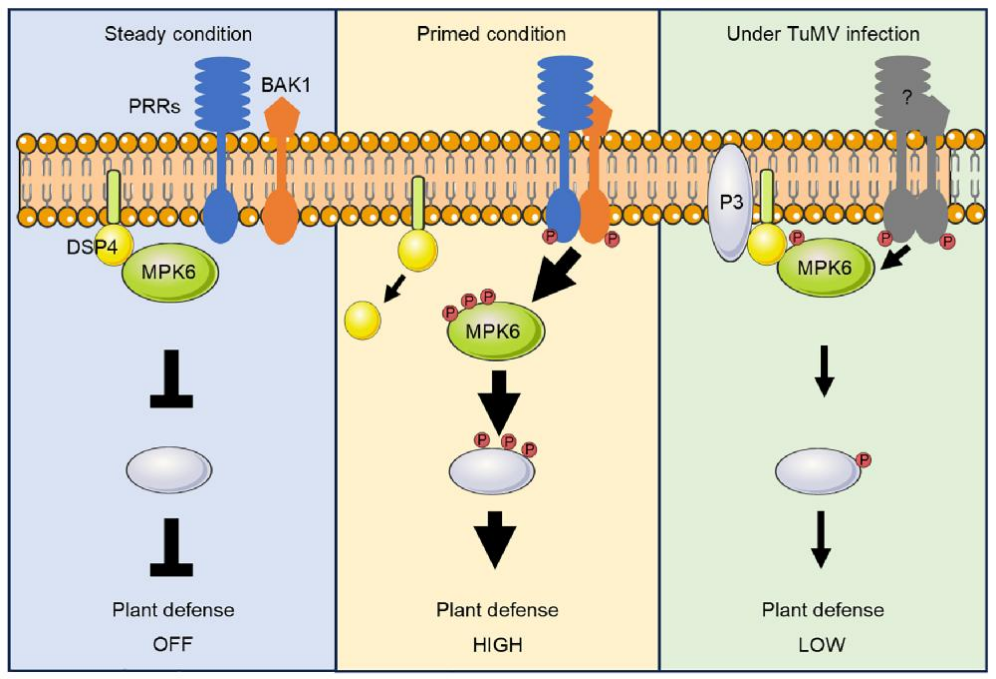
Model proposed by Luan and colleagues describing the regulatory activity of the membrane-bound tyrosine phosphatase DSP4 during basal (steady), active (primed), and viral-infected conditions. Adapted from Figure 7 of Luan et al. (2025), copyright American Society of Biologists.

## Recent related articles in *The Plant Cell*

- Ding et al. (2022) show that the barley MPK3 directly phosphorylates the nucleoprotein of Barley yellow striate mosaic virus to inhibit infection, and they propose a cross-kingdom mechanism that regulates the plant–virus–insect interaction.
- Ma et al. (2025) characterize the activity of OXI1 kinase, which activates MAPKKK5 signaling in response to chitin to activate antifungal defenses.
- Kim et al. (2023) describe the regulation of the MAPKKK5 INTEGRIN-LINKED KINASE 5 (ILK5) by the extracellular ATP receptor P2K1 in response to elicitors of PTI and during infection to *Pseudomonas syringae*.

## Dive Curated Terms

The following phenotypic, genotypic, and functional terms are of significance to the work described in this paper:

MAPKKK5 Gramene: AT5G66850
MAPKKK5 Araport: AT5G66850

MPK6 Gramene: AT2G43790
MPK6 Araport: AT2G43790

MPK3 Gramene: AT3G45640
MPK3 Araport: AT3G45640

ATP CHEBI: CHEBI:15422

DSP4 Gramene: AT3G52180
DSP4 Araport: AT3G52180

OXI1 Gramene: AT3G25250
OXI1 Araport: AT3G25250

## References

[koaf238-B1] Ding ZH, Gao Q, Tong X, Xu WY, Ma L, Zhang ZJ, Wang Y, Wang XB. MAPKs trigger antiviral immunity by directly phosphorylating a rhabdovirus nucleoprotein in plants and insect vectors. Plant Cell. 2022:34(8):3110–3127. 10.1093/plcell/koac143 35567529 PMC9338794

[koaf238-B2] He H, Su J, Shu S, Zhang Y, Ao Y, Liu B, Feng D, Wang J, Wang H. Two homologous putative protein tyrosine phosphatases, OsPFA-DSP2 and AtPFA-DSP4, negatively regulate the pathogen response in transgenic plants. PLoS One. 2012:7(4):e34995. 10.1371/journal.pone.0034995 22514699 PMC3325911

[koaf238-B3] Kim D, Chen D, Ahsan N, Jorge GL, Thelen JJ, Stacey G. The Raf-like MAPKKK INTEGRIN-LINKED KINASE 5 regulates purinergic receptor-mediated innate immunity in Arabidopsis. Plant Cell. 2023:35(5):1572–1592. 10.1093/plcell/koad029 36762404 PMC10118279

[koaf238-B4] Lin L, Luo Z, Yan F, Lu Y, Zheng H, Chen J. Interaction between potyvirus P3 and ribulose-1,5-bisphosphate carboxylase/oxygenase (RubisCO) of host plants. Virus Genes. 2011:43(1):90–92. 10.1007/s11262-011-0596-6 21400205

[koaf238-B5] Luan Y, Jiang X, Wang Y, Chai M, Li F, Wang A, Wu X, Cheng X. A plant RNA virus hijacks a membrane-anchored dual-specificity phosphatase to attenuate MAPK-mediated immunity for robust infection. Plant Cell 2025:37(10):koaf232. 10.1093/plcell/koaf232 40990618

[koaf238-B6] Ma M, Wang P, Chen R, Bai M, He Z, Xiao D, Xu G, Wu H, Zhou JM, Dou D, et al The OXIDATIVE SIGNAL-INDUCIBLE1 kinase regulates plant immunity by linking microbial pattern–induced reactive oxygen species burst to MAP kinase activation. Plant Cell. 2025:37(1):koae311. 10.1093/plcell/koae311 PMC11663599 39566103

[koaf238-B7] Sun T, Zhang Y. MAP kinase cascades in plant development and immune signaling. EMBO Rep. 2022:23(2):e53817. 10.15252/embr.202153817 35041234 PMC8811656

[koaf238-B8] Xin J, Li C, Ning K, Qin Y, Shang JX, Sun Y. AtPFA-DSP3, an atypical dual-specificity protein tyrosine phosphatase, affects salt stress response by modulating MPK3 and MPK6 activity. Plant Cell Environ. 2021:44(5):1534–1548. 10.1111/pce.14002 33464564

